# Reinforcement Learning-Based Multihop Relaying: A Decentralized Q-Learning Approach

**DOI:** 10.3390/e23101310

**Published:** 2021-10-06

**Authors:** Xiaowei Wang, Xin Wang

**Affiliations:** College of Information Engineering, Shanghai Maritime University, Shanghai 201306, China; 202030310238@stu.shmtu.edu.cn

**Keywords:** reinforcement learning, Q-learning, multihop network, relay selection

## Abstract

Conventional optimization-based relay selection for multihop networks cannot resolve the conflict between performance and cost. The optimal selection policy is centralized and requires local channel state information (CSI) of all hops, leading to high computational complexity and signaling overhead. Other optimization-based decentralized policies cause non-negligible performance loss. In this paper, we exploit the benefits of reinforcement learning in relay selection for multihop clustered networks and aim to achieve high performance with limited costs. Multihop relay selection problem is modeled as Markov decision process (MDP) and solved by a decentralized Q-learning scheme with rectified update function. Simulation results show that this scheme achieves near-optimal average end-to-end (E2E) rate. Cost analysis reveals that it also reduces computation complexity and signaling overhead compared with the optimal scheme.

## 1. Introduction

Multihop relaying is believed to extend transmission range and to form the essential communication structure for many practical networks, such as *ad hoc* networks and vehicular networks. In these networks, candidate relays for each hop are often clustered. For example, in vehicular networks, a vehicle gets access to a roadside unit (RSU) with help of multiple relay vehicles which are often geographically clustered. Therefore, judiciously designed relay selection policies guarantee a stable and efficient communication path. The optimal selection policy searches for the best path based on maximization algorithm and inter-cluster channel state information (CSI) of all hops. Its computational complexity and signaling overhead are considerably high. Then, decentralized selection schemes have been proposed to reduce costs by compromising a proportion of performance [1,2,3,4]. Ref. [2] considered clustered multihop networks and proposed a decentralized relay selection scheme which selects a set of relays. This scheme explores multiuser diversity but causes interference, so the size of selected set should be very small. In [3], a decentralized selection scheme is proposed to choose the best relay for each cluster with the consideration of physical-layer security. Another way to design decentralized relay selection is to set a timer for each node within a cluster, which is the reciprocal of CSI. The node whose timer counts to 0 first is selected as the relay. In spite of these academic efforts, satisfactory tradeoff between performance and cost has not been achieved. Therefore, it is meaningful to investigate brand-new decentralized selection scheme which further narrows the performance gap to the optimal policy.

Recently, machine learning has found its extensive applications in optimizations for wireless communications such as antenna selection [5,6], relay selection [7,8,9], and power allocation [10,11]. Learning tools used to solve these problems include supervised learning [5,6,7,8], reinforcement learning [12], neural network (NN) [9], etc. The flourish of learning-based optimization inspires us to exploit new multihop relay selection schemes. Recently, more complex optimization problems have been solved by reinforcement learning in dualhop relay networks, combining relay selection with other techniques such as energy harvesting [13], buffer aided relays [14], device-to-device (D2D) communications [15], access control [16], etc. In [7,8], relay selection for dualhop networks is modeled as multi-class classification and solved by decision tree. However, multihop clustered relaying yields large number of possible paths, which makes classification inefficient. To solve multihop relay selection problem, we will design a novel learning-based scheme. In [17,18], relay selection schemes based on reinforcement learning are proposed for dualhop networks, but these schemes cannot be extended to multihop networks.

In this paper, multihop relay selection is modeled as Markov decision processes (MDP) and solved by reinforcement learning. We propose a Q-learning-based decentralized algorithm which allows each cluster to train its own Q-table and predict relay selection. We aim to reduce computational complexity and signaling overhead while keeping near-optimal average end-to-end (E2E) rate.

## 2. System Model and Optimization-Based Relay Selection

### 2.1. Communication Model

We consider a linear multihop network with a source node (S), a destination node (D) and *M* clusters of relays denoted by $C_{1},\ldots ,C_{M}$. $C_{m}$ consists of $K_{m}$ decode-and-forward (DF) relay nodes denoted by $R_{1}^{m},R_{2}^{m},\ldots ,R_{K_{m}}^{m}$, m=1,…,M. For convenience, we let $C_{0}$ denote S and $C_{M+1}$ denote D. Then, $K_{0}=K_{M+1}=1$. Neither the relays nor S has direct link to D, except the nodes in $C_{M}$. The signal transmitted by S is delivered to D along a path composed of *M* relays selected from the *M* clusters. At the beginning of the multihop transmission, S broadcasts its data to $C_{1}$. One member of $C_{1}$ is selected to receive the data and broadcasts it to $C_{2}$. By this means, the data is relayed until it is received by D.

The wireless channels in the network are assumed to experience independent and identically distributed (i.i.d.) Rayleigh fading. The noise at each receiver is modeled as complex Gaussian random variable with zero mean and variance $\sigma^{2}$. $h_{k^{\prime }k}^{m}$ denotes inter-cluster complex channel coefficients from $R_{k^{\prime }}^{m-1}$ to $R_{k}^{m}$. When m=M+1, $h_{k^{\prime }k}^{M+1}=h_{k^{\prime }D}^{M+1}$.

Assuming that transmitting power of $R_{k^{\prime }}^{m-1}$ is $P_{m-1}$, received signal-to-noise ratio (SNR) of $R_{k}^{m}$ is given by

(1) $$\begin{array}{c} \Gamma_{k^{\prime }k}^{m}=\frac{P_{m-1}{\left|h_{k^{\prime }k}^{m}\right|}^{2}}{\sigma^{2}},\;m=1,\ldots ,M+1. \end{array}$$

E2E SNR and E2E rate of the multihop communication are expressed as

(2) $$\begin{array}{c} \Gamma_{E}=\min_{m}\Gamma_{k^{\prime }k}^{m},\;m=1,\ldots ,M+1 \end{array}$$

and

(3) $$\begin{array}{c} R_{e2e}=\frac{1}{M+1}\log\left(1+\Gamma_{E}\right). \end{array}$$

### 2.2. Optimization-Based Relay Selection

#### 2.2.1. Optimal Selection

In the considered network, there are $\prod_{m}K_{m}$ possible paths. The optimal selection is a centralized maximization-based scheme which chooses the best path. The central controller collects $h_{k^{\prime }k}^{m}$ for all *k*, $k^{\prime }$, *m*, and computes $\Gamma_{k^{\prime }k}^{m}$ and $\Gamma_{E}$ for each path. The optimal policy is to select the path (relay combination) yielding maximum $R_{e2e}$, as described by

(4) $$\begin{array}{ccc} \left(1^{*},2^{*},\ldots ,m^{*},\ldots ,M^{*}\right) & & =\arg\max_{\mathrm{all}\;\mathrm{paths}}R_{e2e}\\ & & =\arg\max_{\mathrm{all}\;\mathrm{paths}}\min_{m}\Gamma_{k^{\prime }k}^{m}. \end{array}$$

Here, $m^{*}$ denotes the index of selected relay of $C_{m}$ and $0^{*}$ represents S.

Selecting the maximum from $\prod_{m}K_{m}$ values is of $O(\prod_{m}K_{m})$ complexity, and requires all inter-cluster CSI. Designing decentralized multihop relay selection schemes will reduce these costs.

#### 2.2.2. Conventional Decentralized Selection

To distribute the computations, the *M* relays are selected separately and successively at each cluster. For 1≤m≤M−1, the conventional decentralized selection policy is described by

(5) $$\begin{array}{c} m^{*}=\arg\max_{1\leq k\leq K_{m}}\Gamma_{{\left(m-1\right)}^{*}k}^{m} \end{array}$$

and for m=M,

(6) $$\begin{array}{c} M^{*}=\arg\max_{1\leq k\leq K_{M}}\min\left\{\Gamma_{{\left(M-1\right)}^{*}k}^{M},\Gamma_{kD}^{M+1}\right\}. \end{array}$$

## 3. Q-Learning-Based Multihop Relaying

In the considered network, a cluster only requires information from the previous and the next clusters. Hence, multihop transmission is naturally Markov. In this section, we model multihop relay selection as MDP and propose a Q-learning-based decentralized relay selection scheme. The scheme is composed of three phases: initialization, training, and prediction. The tasks of training and prediction are decentralized to each cluster. Hence, each cluster, including D, maintains a Q-table and a reward table; Training and prediction are completed in a successive manner from $C_{1}$ to $C_{M}$. The Q-tables are updated for multiple episodes until convergence is reached, and then are used to search for the best relays. First, we provide basic definitions for a standard Q-learning algorithm [19], taking the algorithm on $C_{m}$ for example.

State: $s_{m}$ represents the selected relay node of $C_{m-1}$, which broadcasts the data-carrying signal to $C_{m}$.

Action: $a_{m}$ represents the relay node selected from $C_{m}$ which will receive the signal broadcast by $s_{m}$. For state $s_{m}=R_{k^{\prime }}^{m-1}$, possible actions involve all relay nodes of $C_{m}$, i.e., $a_{m}\in \left\{R_{k}^{m}|k=1,\ldots ,K_{m}\right\}$.

Reward: $r_{m}\left(s_{m},a_{m}\right)$ denotes the reward of state $s_{m}$ if action $a_{m}$ is taken, and is stored in the reward table of $C_{m}$. If $s_{m}=R_{k^{\prime }}^{m-1}$, $a_{m}=R_{k}^{m}$, $r_{m}\left(s_{m},a_{m}\right)$ is defined by the SNR from $s_{m}$ to $a_{m}$, i.e.

(7) $$\begin{array}{c} r_{m}\left(R_{k^{\prime }}^{m-1},R_{k}^{m}\right)=\Gamma_{k^{\prime }k}^{m}. \end{array}$$

(8) $$\begin{array}{ccc} Q_{m}\left(s_{m},a_{m}\right)\;\;\;\;\;\; & & \;\;\;\;\;=\left\{\begin{array}{cc} \left(1-\alpha \right)Q_{m}\left(s_{m},a_{m}\right)+\alpha \left(r_{m+1}+\gamma Q_{\max}\left(m+1\right)\right), & m\leq M,r_{m}\left(s_{m},a_{m}\right)>r_{m+1}\\ \left(1-\alpha \right)Q_{m}\left(s_{m},a_{m}\right)+\alpha \left(r_{m}\left(s_{m},a_{m}\right)+\gamma Q_{\max}\left(m+1\right)\right), & m\leq M,r_{m}\left(s_{m},a_{m}\right)<r_{m+1}\\ r_{m}\left(s_{m},a_{m}\right), & m=M+1. \end{array}\right. \end{array}$$

Q-value: $Q_{m}\left(s_{m},a_{m}\right)$ denotes the Q-value for a given state-action pair $\left(s_{m},a_{m}\right)$, which is defined to evaluate the accumulated value of $s_{m}$. $Q_{m}\left(s_{m},a_{m}\right)$ is stored in the Q-table of $C_{m}$ and is obtained by iterative updating.

**Figure 1:**
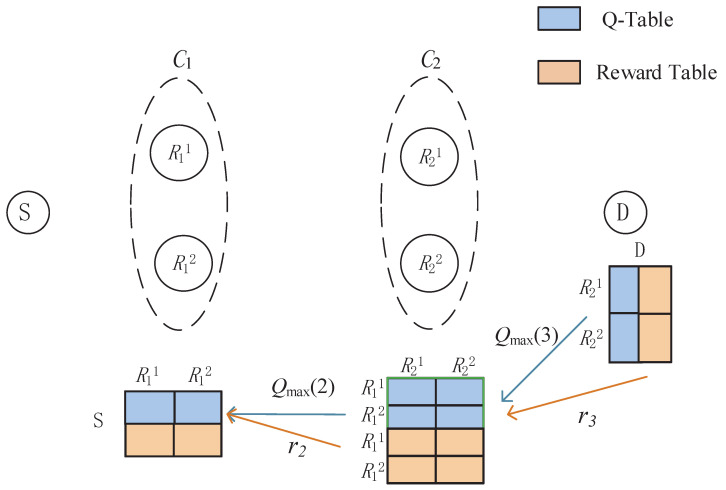
Q-learning-based multihop relaying.

System parameters include learning rate α, discount factor γ, and error threshold of convergence ε. A brief illustration of this scheme is given in Figure 1.

### 3.1. Initialization

The reward table and Q-table of $C_{m}$ are initialized by $K_{m-1}\times K_{m}$ tables, m=0,1,…,M+1. The reward table stores the rewards of all possible state-action pairs. To initialize the reward table, $C_{m}$ estimates CSI of the channels from each node of $C_{m-1}$ to each node of its own, and calculates reward values using (7). The Q-table stores Q-values of all $\left(s_{m},a_{m}\right)$ pairs. Each row represents a node in $C_{m-1}$ and each column represents a node in $C_{m}$. All Q-values of tables from $C_{1}$ to $C_{M}$ are initialized to be 0. The Q-table of D is fixed and duplicated from D’s reward table. The reward table and Q-table of $C_{m}$ are described by Table 1 and Table 2.

### 3.2. Training

Training phase is iteratively updating the Q-tables of each cluster in a successive manner for multiple episodes until convergence is reached. The key issue of training is the update function. If we adopt the standard update function given by [19], only the reward of current state is involved, i.e. the rate of current hop. However, in DF multihop relaying, the rate of an individual hop cannot contribute to the calculation of end-to-end rate if the rate of another hop is even smaller. Therefore, the update function should take into account all hops, which is not economical. We predict that the standard update function will not yield high performance. Instead, we revise the standard update function and make it choose the larger one between the rewards of current hop and the next hop. This means that the update function can eventually maximize the end-to-end rate. The new update function is given by (8).

In the beginning of the *n*th updating episode, $s_{1}=S$ and $a_{1}$ is randomly selected from $C_{1}$. $C_{2}$ chooses the best action, $a_{2}^{\prime }$, by

(9) $$\begin{array}{c} a_{m+1}^{\prime }=\arg\max_{a_{m+1}}Q_{m+1}\left(a_{m},a_{m+1}\right), \end{array}$$

and computes the Q-value and reward of $a_{2}^{\prime }$ by

(10) $$\begin{array}{c} Q_{\max}\left(m+1\right)=Q_{m+1}\left(a_{m},a_{m+1}^{\prime }\right), \end{array}$$

(11) $$\begin{array}{c} r_{m+1}=r_{m+1}\left(a_{m},a_{m+1}^{\prime }\right). \end{array}$$

Then, $Q_{\max}\left(2\right)$ and $r_{2}$ are sent back to $C_{1}$, and are used to update $Q_{1}\left(S,a_{1}\right)$ by (8).

For $C_{2}$, $s_{2}=a_{1}$, and $a_{2}$ is randomly selected from $C_{2}$. The rest procedures are the same as $C_{1}$, and $Q_{2}\left(s_{2},a_{2}\right)$ is updated. The above updating procedure for a single cluster is repeated to each of the following clusters to finish this episode. If the error of E2E rate is lower than ε, the Q-tables are converged and training is completed. Else, a new episode of updating begins.

### 3.3. Prediction

Successively from $C_{1}$ to $C_{m}$, each cluster searches its Q-table and selects the best relay. First, $C_{1}$ searches its single-row Q-table for the action with maximum Q-value and selects it as the best relay. This selection policy is described by

(12) $$\begin{array}{c} m^{*}=\arg\max_{a\in C_{m}}Q_{m}\left({\left(m-1\right)}^{*},a\right),\;m=1,\ldots ,M. \end{array}$$

Notified of $1^{*}$, $C_{2}$ searches the corresponding row of $1^{*}$ from its Q-table and obtains $2^{*}$. After all clusters complete their relay selection, a multihop path is established through which the source data is delivered to D.

The proposed Q-learning-based relay selection scheme for multihop clustered networks is summarized in Algorithm 1.

**Algorithm 1.** Q-learning-based multihop relay selection. 
**Phase 1. Initialization**

**for**

m=1:M+1

 $C_{m}$ evaluates and collects $h_{k^{\prime }k}^{m}$ for all $k^{\prime }$ and *k* $C_{m}$ generates a $K_{m-1}\times K_{m}$ reward table and initializes it by (7) $C_{m}$ generates a $K_{m-1}\times K_{m}$ Q-table. **if**   m=1,...,M, all Q-values are set to be 0. **else**   Q-values are duplicated from reward table.
**end**

**Phase 2. Training**


$n=1,R_{0}=0$

while (1)

$s_{1}=S$


**for**

m=1:M

 $C_{m}$ randomly selects an action $a_{m}$ from $C_{m}$ Request $Q_{\max}\left(m+1\right)$ and $r_{m+1}$ from $C_{m+1}$ Update the $Q_{m}\left(s_{m},a_{m}\right)$ using (8) $s_{m+1}=a_{m+1}^{\prime }$
**end**
Compute end-to-end rate $R_{n}$
**If**

$R_{n}-R_{n-1}<\epsilon$

 break
**else**
 n=n+1
**end**

**end**

**Phase 3. Prediction**
Let $0^{*}=S$
**for**

m=1:M

 $C_{m}$ searches in its Q-table and selects the best relay $m^{*}$ using (12) Notify $m^{*}$ to $C_{m+1}$ $R_{m^{*}}^{m}$ receives the signal transmitted from $R_{{\left(m-1\right)}^{*}}^{m-1}$, and broadcasts it to $C_{m+1}$
**end**


## 4. Performance Evaluation

### 4.1. Simulation Results

We simulate a multihop network with *M* clusters and each cluster contains equally *K* relays. Average E2E rate is calculated as the performance metric. Simulation parameters are given in Table 3. We first examine the convergence of the Q-learning-based relay selection. Then, the scheme is compared with optimal scheme given by (4) and conventional decentralized scheme described by (5) and (6).

**Figure 2:**
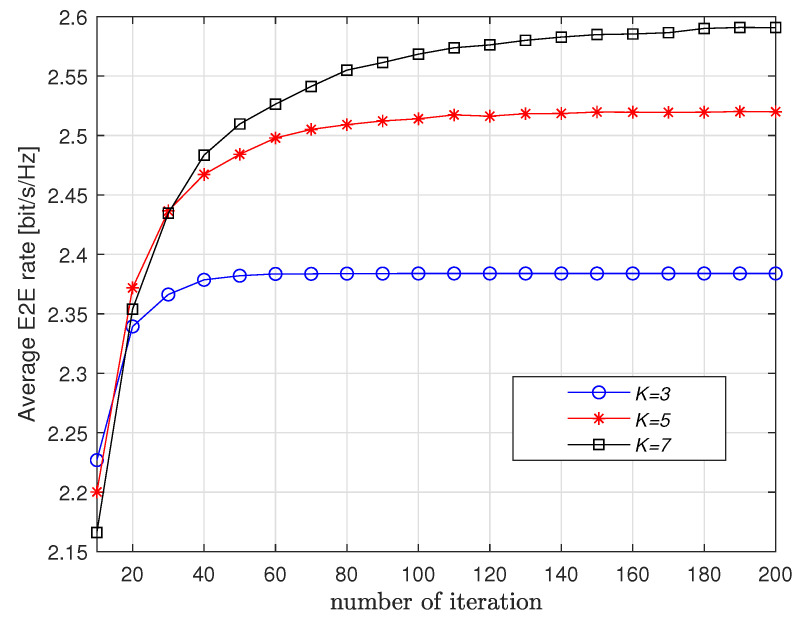
Convergence vs. *K* when M=3.

**Figure 3:**
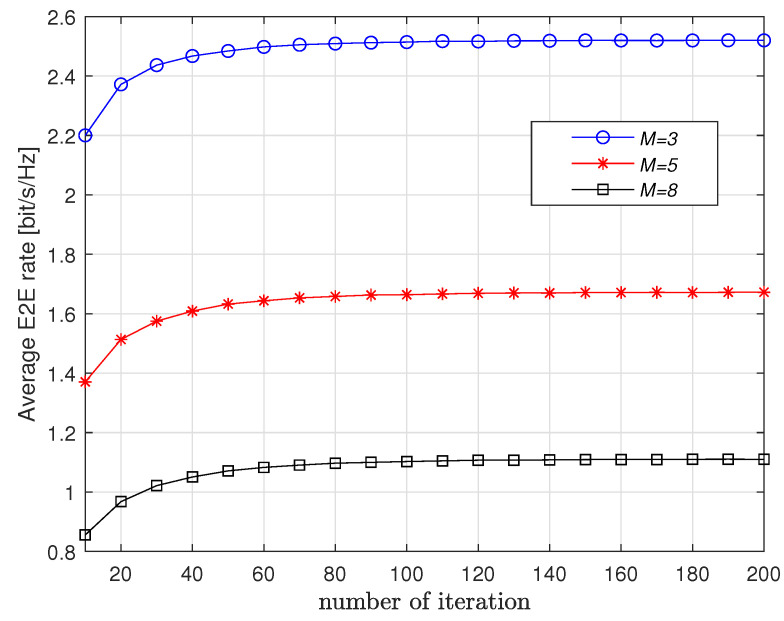
Convergence vs. *M* when K=5.

First, Figure 2 and Figure 3 show the convergence of the proposed Q-learning scheme with respect to *K* and *M*. It is observed from Figure 2 that more iterations are needed for convergence if *K* increases. When *K* is fixed, we observe from Figure 3 that *M* has little impact on the number of iterations for convergence. This means that the proposed scheme applies to long route.

**Figure 4:**
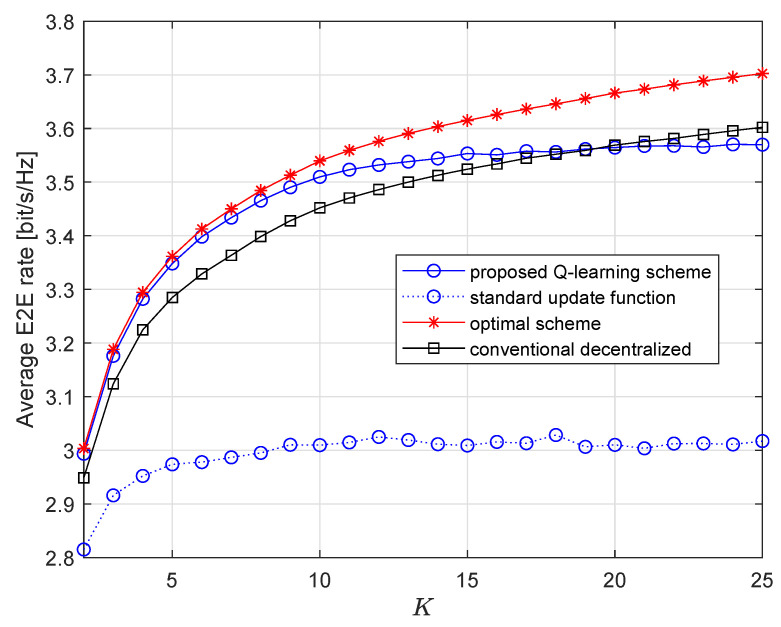
Average E2E rate vs. *K* when M=2.

Figure 4 shows average E2E rates of the three schemes with respect to *K*. The first observation is that the curve of Q-learning scheme is very close to the curve of the optimal scheme, especially when *K* is small. The gap grows larger when *K* increases, which indicates that Q-learning scheme cannot consistently benefit from growing *K*. Without designed update function, the Q-learning scheme achieves the lowest E2E rate and cannot benefit from growing *K*. Another important observation is that when K≤19, Q-learning scheme clearly outperforms the conventional decentralized scheme. After K=19, the conventional decentralized scheme yields better performance. This is because great *K* yields large action space, and Q-learning cannot work well with large action space. This issue can be easily avoided because larger *K* also increases computational complexity. So, normally cluster size should be controlled.

**Figure 5:**
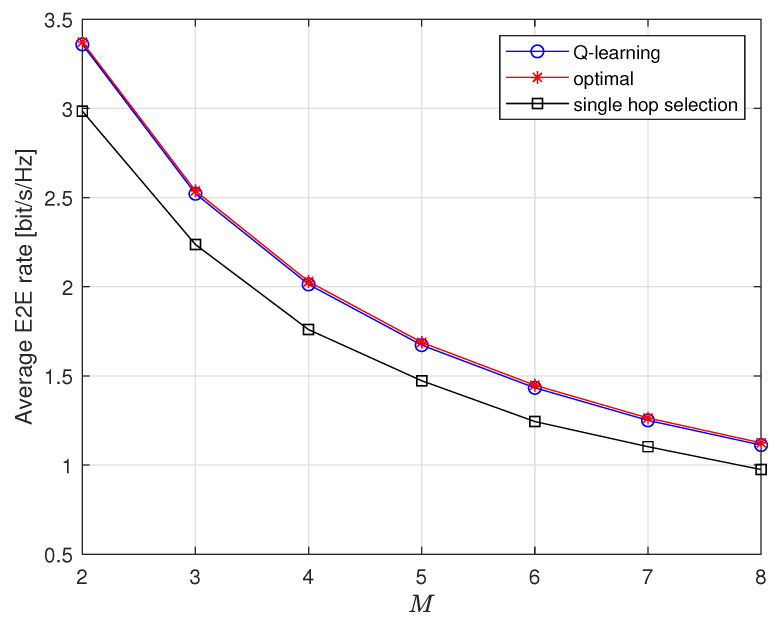
Average E2E rate vs. *M* when K=5.

Figure 5 illustrates average E2E rates with respect to *M*. As *M* grows larger, average E2E rates of all three schemes decrease because E2E rate is bounded by the worst hop. We also observe that the curve of Q-learning scheme is very close to optimal scheme. Moreover, Q-learning scheme clearly outperforms conventional decentralized scheme. The advantage of Q-learning scheme keeps unchanged when *M* increases.

From above figures, we summarize that the proposed Q-learning scheme achieves near-optimal E2E rate. To take the best advantage of it, the proposed scheme is better applied to multihop linear networks with moderate cluster size.

### 4.2. Cost Analysis

#### 4.2.1. Computational Complexity

The optimal policy selects the path with maximum E2E rate among all $K^{M}$ paths, leading to computational complexity of $O(K^{M})$. In the proposed Q-learning scheme, the complexities of initialization and prediction are $O(K^{2})$ and O(K). The main part of training phase is iterative updating of Q-tables, leading to complexity of $O(MK\log\frac{1}{\varepsilon })$. In most practical networks, Q-learning scheme is superior to the optimal scheme.

#### 4.2.2. CSI Amount

The optimal scheme is centralized and requires CSI of all $\left(M-1\right)K^{2}+2K$ inter-cluster links reported to the central controller. In the proposed Q-learning scheme, CSI of $\left(M-1\right)K^{2}+2K$ inter-cluster links is only estimated and collected locally between adjacent clusters. Thus, total energy consumed and interference to other transmissions caused by signaling are greatly reduced. In each iterative update, each cluster requires only two values, $Q_{\max}\left(m+1\right)$ and $r_{m+1}$, from the next cluster to update its Q-table. This causes extra communication costs. Moreover, each cluster transmits the selected action $a_{m}$ to the next cluster, costing only $\log_{2}K$ bits.

Signaling overhead of multihop networks is mainly due to CSI collection. To evaluate CSI amount, we propose a calculating method which takes into account both the number of channels to be estimated and the length of CSI transmission route. We suppose that the central controller is located at the middle cluster of the multihop path. In the optimal scheme, the CSI of faraway clusters is delivered to the central controller via multihop transmission. Thus, we calculate the weighted sum CSI amount, and let the weights be the numbers of hops needed to collect the CSI. The Q-learning-based scheme and conventional decentralized scheme only require CSI transmission between adjacent clusters, so all weights are 1. The weighted CSI amount of the optimal scheme is calculated by (13). It is not difficult to prove that C(M,K) is always greater than $\left(M-1\right)K^{2}+2K$, CSI amount of the Q-learning-based scheme, for all values of *M* and *K*. The three schemes are compared in Table 4.

(13) $$\begin{array}{c} C\left(M,K\right)=\left\{\begin{array}{cc} & \frac{\left(M^{2}-1\right)K^{2}}{4}+\left(M+1\right)K,\;M=3,5,7,...\\ & \frac{M^{2}K^{2}}{4}+\left(M+1\right)K,\;\;\;\;\;\;\;\;\;\;M=2,4,6,.... \end{array}\right. \end{array}$$

## 5. Conclusions

In this paper, we have proposed a decentralized Q-learning-based relay selection scheme for multihop clustered networks. The scheme is composed of three phases: initialization, training, and prediction. A new update function for Q-values is designed to promote prediction performance. Simulation results show that the proposed Q-learning scheme achieves near-optimal performance and outperforms conventional decentralized scheme in terms of average E2E rate. The advantages of Q-learning scheme also lie in lower computational complexity and smaller cost for collecting CSI.

## Figures and Tables

**Table 1:** Reward table of the *m*th cluster.

$s_{m}\setminus a_{m}$	$R_{1}^{m}$	$R_{2}^{m}$	...	$R_{K_{m}}^{m}$
$R_{1}^{m-1}$	$\Gamma_{11}^{m}$	$\Gamma_{12}^{m}$	...	$\Gamma_{1K_{m}}^{m}$
$R_{2}^{m-1}$	$\Gamma_{21}^{m}$	$\Gamma_{22}^{m}$	...	$\Gamma_{2K_{m}}^{m}$
...	...	...	...	...
$R_{K_{m-1}}^{m-1}$	$\Gamma_{K_{m-1}1}^{m}$	$\Gamma_{K_{m-1}2}^{m}$	...	$\Gamma_{K_{m-1}K_{m}}^{m}$

**Table 2:** Q-table of the *m*th cluster.

$s_{m}\setminus a_{m}$	$R_{1}^{m}$	$R_{2}^{m}$	...	$R_{K_{m}}^{m}$
$R_{1}^{m-1}$	$Q_{m}\left(R_{1}^{m-1},R_{1}^{m}\right)$	$Q_{m}\left(R_{1}^{m-1},R_{2}^{m}\right)$	...	$Q_{m}\left(R_{1}^{m-1},R_{K_{m}}^{m}\right)$
$R_{2}^{m-1}$	$Q_{m}\left(R_{2}^{m-1},R_{1}^{m}\right)$	$Q_{m}\left(R_{2}^{m-1},R_{2}^{m}\right)$	...	$Q_{m}\left(R_{2}^{m-1},R_{K_{m}}^{m}\right)$
...	...	...	...	...
$R_{K_{m-1}}^{m-1}$	$Q_{m}\left(R_{K_{m-1}}^{m-1},R_{1}^{m}\right)$	$Q_{m}\left(R_{K_{m-1}}^{m-1},R_{2}^{m}\right)$	...	$Q_{m}\left(R_{K_{m-1}}^{m-1},R_{K_{m}}^{m}\right)$

**Table 3:** Simulation parameters.

Transmit Power $P_{m}$	30 dB
noise power $\sigma^{2}$	1
learning rate α	1
discount factor γ	0.4
fading parameter $E[|h_{k^{\prime }k}^{m}{|}^{2}]$	1
convergence threshold ε	$10^{-3}$

**Table 4:** Comparison of the Q-learning-based scheme with benchmark schemes.

	Complexity	CSI Amount	E2E Rate
optimal	$O(K^{M})$	C(M,K)	optimal
conventional decentralized	O(MK)	(M+1)K	below optimal
Q-learning	$O(MK\log\frac{1}{\varepsilon })$	$\left(M-1\right)K^{2}+2K$	near optimal

## Data Availability

Not applicable.
